# Different People, Different Outcomes: Assessing Genetic Susceptibility to Lead Exposures

**DOI:** 10.1289/ehp.124-A131

**Published:** 2016-07-01

**Authors:** Julia R. Barrett

**Affiliations:** Julia R. Barrett, MS, ELS, a Madison, WI–based science writer and editor, is a member of the National Association of Science Writers and the Board of Editors in the Life Sciences.

Prenatal and early-life exposures to lead have long been known to cause an array of adverse developmental effects in children.^1^ However, the variety of responses to lead, even with exposures, suggests that an individual’s genetic background might influence how lead toxicity manifests.^2^ ^,^ ^3^ ^,^ ^4^ A new study in fruit flies scrutinizes susceptibility on the genetic level and highlights genes that might help shape individual responses.^5^

“There are several problems in trying to find out variations and susceptibility to lead in human populations,” says study coauthor Robert Anholt, a professor of biological sciences at North Carolina State University. Uncontrolled genetic backgrounds, mixtures of contaminants in the environment, and additional factors such as diet and smoking all muddy the waters; in addition, lead’s effects may not be apparent until years after the exposure.^1^

**Figure:**
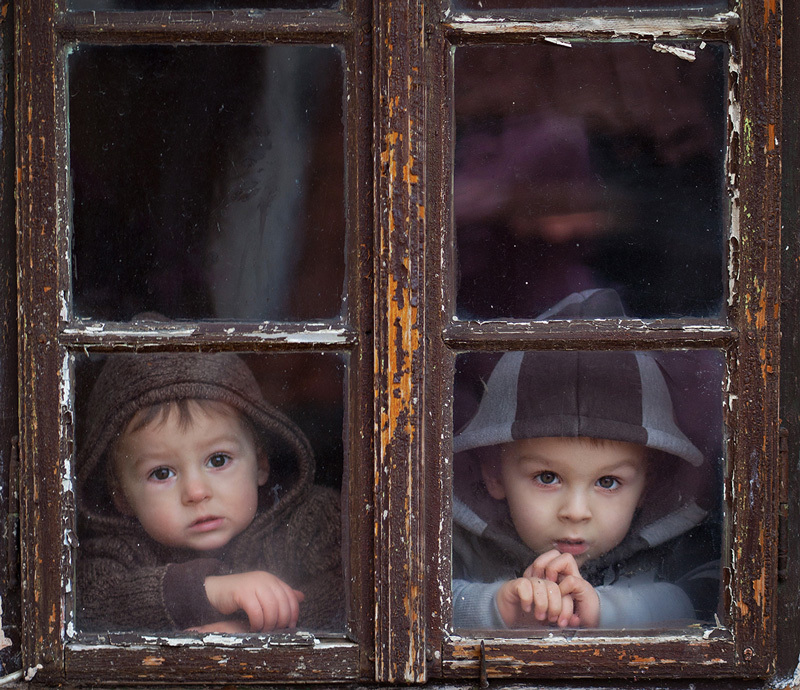
Similar lead exposures can cause very different responses in different people. An individual’s genetic makeup may be one reason why. © Getty/Tatyana Tomsickova

To offset some of the complexity, model systems such as fruit flies (*Drosophila melanogaster*) are used to identify candidate genes, which can then be studied more closely in humans.^5^ “*Drosophila* may seem a little far-fetched, but they are actually a really good system,” says Anholt. Many *Drosophila* genes have human orthologs; that is, they perform the same function in both species.^6^ Anholt calls fruit flies “probably the most powerful, most versatile genetic model we have.”

For the current study the researchers used the *Drosophila melanogaster* Genetic Reference Panel. This unique collection of more than 200 *Drosophila* lines (or varieties) represents varied genetic backgrounds, with all individuals within the same line being nearly identical genetically.^6^ The researchers placed 50 larvae from each of 200 selected lines on control or lead-spiked medium. The timing of their development was based on how many days passed until adult flies emerged, and viability was assessed by counting the number of larvae that survived to adulthood.

Twenty adult flies of each sex were then randomly selected from each line and individually placed in tubes. There, their activity was measured based on how many times they crossed an infrared beam. Based on differences in the traits of viability, development, and activity between the lines, the researchers conducted genome-wide association analyses to identify candidate genes potentially related to those traits. The researchers confirmed the results of the analyses by rearing 20 mutant fruit fly lines on control and lead-supplemented media. Each line contained a specific variant of a single candidate gene that had been implicated in altered activity and development time. Finally, human orthologs of many of the candidate *Drosophila* genes were identified.

In general, lead-exposed flies developed more slowly and were less likely to survive than control flies, although these factors varied greatly among both control and lead-exposed lines. Of the 200 lines used, only 166 produced enough adults for activity measurements. Again, extensive variation occurred among lines and among the lead-exposed flies, with some becoming more active and others becoming less active. The majority of the implicated genes were associated with nervous system development and function.

“Some genetic factors may modulate the sensitivity to lead, and there have been several genes that have been identified that can influence the accumulation and toxicokinetics of lead in humans^7^ ^,^ ^8^ ^,^ ^9^ ^,^ ^10^,” says Jay Schneider, a professor of pathology, anatomy, and cell biology at Thomas Jefferson University, who was not involved in the current study. These genes are not targets of lead toxicity; rather, they influence the absorption, distribution, metabolism, and excretion of lead.^3^ ^,^ ^4^ Other genes might further influence how cells respond to lead.^2^ ^,^ ^5^

This study was not intended to gauge the level at which lead is toxic, and it did not reveal the mechanisms by which lead causes adverse effects. However, if these results are echoed in humans, they could help us understand why and how the effects from lead exposure are expressed differently in different people, says Schneider. “This kind of work shows how much we still have to learn about the potential effects that lead has on the body,” he says. “We know a lot, but there’s still a lot we don’t know.”
